# The Impact of CYP3A4*22 on Tacrolimus Pharmacokinetics and Outcome in Clinical Practice at a Single Kidney Transplant Center

**DOI:** 10.3389/fgene.2019.00871

**Published:** 2019-09-26

**Authors:** Emaad Abdel-Kahaar, Stefan Winter, Roman Tremmel, Elke Schaeffeler, Christoph J. Olbricht, Eberhard Wieland, Matthias Schwab, Maria Shipkova, Simon U. Jaeger

**Affiliations:** ^1^Institute of Pharmacology of Natural Products and Clinical Pharmacology, University of Ulm, Ulm, Germany; ^2^Department of Pharmacology, Qena Faculty of Medicine, South Valley University, Qena, Egypt; ^3^Dr. Margarete Fischer-Bosch Institute of Clinical Pharmacology, Stuttgart, Germany; ^4^University of Tübingen, Tübingen, Germany; ^5^Transplant Center, Klinikum Stuttgart, Stuttgart, Germany; ^6^Central Institute for Clinical Chemistry and Laboratory Medicine, Klinikum Stuttgart, Stuttgart, Germany; ^7^Departments of Clinical Pharmacology, Pharmacy and Biochemistry, University of Tübingen, Tübingen, Germany

**Keywords:** tacrolimus, renal transplantation, pharmacogenetics, *CYP3A5*, CYP3A4*22, *ABCB1*, therapeutic drug monitoring

## Abstract

**Background:** Although there is evidence that the CYP3A4*22 variant should be considered in tacrolimus dosing in renal transplantation, its impact beyond tacrolimus dose requirements remains controversial.

**Methods:** In a cohort of 121 kidney transplant recipients, we analyzed the CYP3A4*1B, CYP3A4*22, and CYP3A5*3 alleles and the *ABCB1* variants *1236C>T*, *2677G>T/A*, and *3435C>T* for their impact on exposure and dose requirement. Relevant clinical outcome measures such as acute rejection within the first year after transplantation, delayed graft function, and renal function at discharge (estimated glomerular filtration rate) were evaluated.

**Results:** Extensive metabolizer (n = 17, CYP3A4*1/*1 carriers with at least one CYP3A5*1 allele) showed significantly higher tacrolimus dose requirement (P = 0.004) compared with both intermediate metabolizer (IM, n = 93, CYP3A5*3/*3 plus CYP3A4*1/*1 or CYP3A4*22 carriers plus one CYP3A5*1 allele), and poor metabolizer (n = 11, CYP3A4*22 allele in combination with CYP3A5*3/*3) after onset of therapy. Significantly higher dose requirement was observed in CYP3A5 expressers (P = 0.046) compared with non-expressers again at onset of therapy. Using the log additive genetic model, the area under the curve for the total observation period up to 16 days was significantly associated with the CYP3A5*3 genotype (P = 3.34 × 10^-4^) as well as with the IM or extensive metabolizer phenotype (P = 1.54 × 10^-4^), even after adjustment for multiple testing. Heterozygous carriers for CYP3A4*22 showed significantly higher areas under the curve than the CYP3A4*1/*1 genotype in the second week post-transplantation (adjusted P = 0.016). Regarding clinical outcomes, acute rejection was significantly associated with human leukocyte antigen mismatch (≥3 alleles; OR = 12.14, 95% CI 1.76, 525.21, P = 0.019 after correction for multiple testing). Graft recipients from deceased donors showed higher incidende of delayed graft function (OR 7.15, 95% CI 2.23, 30.46, adjusted P = 0.0008) and a lower estimated glomerular filtration rate at discharge (P = 0.0001). Tested *CYP3A4* or *CYP3A5* variants did not show any effects on clinical outcome parameters. *ABCB1* variants did neither impact on pharmacokinetics nor on clinical endpoints.

**Conclusion:** At our transplantation center, both CYP3A5*3 and, to a lesser extent, CYP3A4*22 affect tacrolimus pharmacokinetics early after onset of therapy with consequences for steady-state treatment in routine clinical practice.

## Introduction

Tacrolimus, a macrolide isolated from *Streptomyces tsukubaensis*, is an inhibitor of T cell proliferation widely used in solid organ transplantation. After kidney transplantation, it is part of the maintenance immunosuppressive regimen including mycophenolic acid and corticosteroids (Kasiske et al., 2010).

Tacrolimus dosing remains a clinical challenge, as its complex pharmacokinetics leads to large interindividual variability in blood levels, with drug toxicity or insufficient immunosuppression being the consequence. Although strategies to determine the optimal starting dose of tacrolimus have been explored, including algorithms for tacrolimus clearance (Passey et al., 2012), initial dosing is still guided by body weight in clinical practice.

The impact of pharmacogenetics on tacrolimus metabolism has been extensively studied. The influence of CYP3A5 expresser/non-expresser status on pharmacokinetics of tacrolimus is well established, and dosing recommendations have been published [e.g., Guideline of the Clinical Pharmacogenetics Implementation Consortium (Birdwell et al., 2015) and the Recommendations of the Dutch Pharmacogenetic Working Group (Dutch Pharmacogenetics Working Group, 2018)].

However, the effect of genotype-adjusted dosing on clinically important outcome measures [e.g., acute rejection (AR)] is still a matter of discussion. AR is regarded as one of the strongest predictors of allograft survival and thus a clinically critical outcome parameter (Matas et al., 1994). A meta-analysis published in 2015 concluded that CYP3A5 non-expressers have a significantly increased risk for transplant rejection (Rojas et al., 2015), but a prospective randomized trial failed to demonstrate a benefit in terms of AR, nephrotoxicity, or survival (Thervet et al., 2010). Also, *CYP3A5-*guided dosing did not lower the incidence of AR in another recent prospective investigation (Shuker et al., 2016). The absence of consistent effects on clinical outcomes found for variation in *CYP3A5* mandates the search for additional genetic or nongenetic factors. Many variants of the tacrolimus-metabolizing enzyme *CYP3A4* have been reported (https://www.pharmvar.org/htdocs/archive/cyp3a4.htm), but the frequency distribution of these variants is usually very low, and functional consequences are missing. However, the CYP3A4*22 allele, an intronic base change from G to A (rs35599367), has been recently correlated with reduced expression of the CYP3A4 enzyme (Wang et al., 2011) and associated with alteration of the pharmacokinetics of several CYP3A4 drugs like tacrolimus, cyclosporine, and statins (Elens et al., 2013).

Regarding pharmacokinetics, the high interindividual variability in tacrolimus trough levels (C_0_) even after including *CYP3A5* genetics is not sufficiently explained as well. For several variants in drug-metabolizing enzymes or transporters, significant associations with tacrolimus C_0_ levels have been reported. However, in a validation study, a panel of 44 variants in selected candidate genes associated with tacrolimus pharmacokinetics was investigated, and variants in *CYP3A5* (including others that are in strong linkage disequilibrium with CYP3A5*3) and the CYP3A4*22 allele hold true for validation (Oetting et al., 2018a). In line with these results, a genome-wide association study comprising 1,345 European Americans (a sub-cohort from the DeKAF genomics study) carrying *CYP3A5* loss-of-function alleles (*3,*6, or *7) found an additional association of the CYP3A4*22 allele with tacrolimus C_0_ levels (Oetting et al., 2018b).

Moreover, heterozygous variant carriers for CYP3A4*22 have been shown to require lower mean tacrolimus doses and have an increased risk of supra-therapeutic tacrolimus levels (Elens et al., 2011a; Pallet et al., 2015). However, if additive information on CYP3A4*22 carrier status does improve the prediction of tacrolimus levels and dose requirements in a clinically significant way, it is still a matter of debate (Elens et al., 2011a; Moes et al., 2014; Elens and Haufroid, 2017; Lloberas et al., 2017).

Thus, the aim of this retrospective single-center study was to further elucidate the impact of the CYP3A4*22 variant on tacrolimus dose requirement and blood levels as well as its impact on AR in the first year after transplantation, delayed graft function (DGF), and renal function upon discharge. We report on 121 kidney transplant recipients whose tacrolimus levels have been closely monitored in the early period after transplantation. In addition to the *CYP* variants, we selected three polymorphisms of *ABCB1* (encoding P-glycoprotein), since contradictory data regarding tacrolimus pharmacokinetics and *ABCB1* genetics in renal transplant patients (reviewed by Tron et al., 2018) have been reported.

## Patients and Methods

### Patient Population

Data from 121 patients who underwent kidney transplantation between 2009 and 2015 at the Transplantation Center of Klinikum Stuttgart, Germany, were available for analysis. The study was approved by the ethics committee of University Hospital, Tuebingen, Germany (616/2013BO2). All patients signed a written informed consent. As induction therapy, 86% (104/121) of kidney transplant recipients received the interleukin-2 receptor antagonist basiliximab, while the remaining 14% received thymoglobulin due to high immunologic risk. The immunosuppressive maintenance therapy after renal transplantation at the transplantation center at Klinikum Stuttgart, Germany is a triple therapy of tacrolimus, mycophenolate mofetil, and prednisolone. Tacrolimus therapy is started at the day of renal transplantation with a dosage of 0.1 mg/kg/day orally and subsequently adjusted to achieve a target tacrolimus C_0_ concentration of 6 to 8 µg/l in the first 3 months after transplantation. Thereafter, the target C_0_ level is 4 to 6 µg/l. The daily dosage of tacrolimus until day 16 after transplantation and at discharge of the patient from the hospital was considered for pharmacogenetic analyses. Mycophenolate sodium 720 mg was given twice a day during the first 3 months starting from day 1. Prednisolone was uniformly administered to all patients according to the therapy protocol, with intravenous 250 mg peri-operatively, and then orally as 0.5 mg per kg body weight until day 14, then continued with 20 mg/day and tapering. Moreover, all recipients receive as part of the standard treatment protocol proton-pump-inhibitors, colecalciferol plus calcium carbonate, cotrimoxazole, and, for the first 4 days, piperacillin/tazobactam intravenously. Depending on the cytomegalovirus (CMV) status, antiviral prophylaxis with valgancyclovir is administered only to CMV-negative recipients receiving a CMV positive graft. Demographic characteristics of the study cohort are shown in **Table 1**.

**Table 1:** Demographic and clinical characteristics of the study cohort.

Total number of patients	121
Sex of patients (male/female)	77 (64%)/44(36%)
Age of patients (years, median/range)	55 (15–77)
Weight (kg, median/range)	76 (42–118)
Pre-emptive transplantation before dialysis	10 (8%)
Re-transplantation	22 (18%)
Living/deceased donor	52(43%)/69(57%)
Induction therapy	
Basiliximab	104 (86%)
Thymoglobulin	17 (14%)
Age of donors (years, median/range)	56 (19–88)
AB0 incompatibility	15 (12%)
HLA mismatches (A, B, DR, median/range)	3 (0–6)
Panel reactive antibodies >10%	22 (18%)
Cold ischemia time (min, median/range)	467 (39–2,113)
Warm ischemia time (min, median/range)	45 (21–86)
Cytomegalovirus (antibody status)	
Donor negative/Recipient negative	22
Donor negative/Recipient positive	33
Donor positive//Recipient negative	16 (12.4%)
Donor positive/Recipient positive	49
Status not available	1
Underlying disease	
Chronic renal failure, etiology uncertain	36
Polycystic kidney disease	24
IgA nephropathy (proven by immunofluorescence)	18
Pyelonephritis/interstitial nephritis due to vesico-ureteral reflux	7
Glomerulonephritis	11
Lupus erythematosus related glomerulonephritis	4
Alport’s syndrome	3
Wegener’s granulomatosis	2
Tubulointerstitial nephritis (not pyelonephritis related)	2
Malignant hypertension	2
Focal segmental glomerulosclerosis with nephrotic syndrome	2
Diabetes type II	2
Congenital renal disorders	3
Rapidly progressive GN	1
Pyelonephritis/interstitial nephritis due to congenital malformation	1
Oligomeganephronic hypoplasia	1
Nephropathy due to analgesic drugs	1
Henoch-Schönlein purpura	1

### Genotyping

Genomic DNA was isolated from 200-µl ethylenediaminetetraacetic acid whole blood using QIAamp DNA Blood Mini Kit (Qiagen, Hilden, Germany) according to the manufacturer’s protocol. For genotyping, the following variants were selected: CYP3A4*1B (rs2740574), CYP3A4*22 (rs35599367), and CYP3A5*3 (rs776746) and the three most relevant *ABCB1* variants 1236C>T (rs1128503), 2677G>T,A (rs2032582), and 3435C>T (rs1045642). Genotyping was performed using TaqMan assays on 7900HT Fast Real-Time PCR System and the allelic discrimination method according to the manufacturer’s instructions (Applied Biosystems, Darmstadt, Germany). CYP3A4*1: assay C___1837671_50; CYP3A4*22: assay C__59013445_10; CYP3A5*3: assay C__26201809_30; *ABCB1*1236C>T, assay C___7586662_10; triallelic *ABCB1*2677: assays C_11711720D_40 (for T allele) and C_11711720C_30 (for A allele); *ABCB1*3435C>T: assay C___7586657_20).

As described previously (Elens et al., 2011b), the combined *CYP3A4/5* genotypes were defined as follows: poor metabolizers (PM, patients carrying the CYP3A4*22 allele in combination with homozygosity for CYP3A5*3), intermediate metabolizer (IM, CYP3A5*3/*3 plus CYP3A4*1/*1 or CYP3A4*22 carriers plus one CYP3A5*1 allele), and extensive metabolizer (EM, CYP3A4*1/*1 with at least one CYP3A5*1 allele).

### Tacrolimus Blood Levels

Trough concentrations C_0_ (nanogram per milliliter) available from the first 2 weeks after transplantation or until the patient was discharged were retrospectively gathered from patient files. Tacrolimus concentrations are measured in whole blood by a validated liquid chromatography with tandem mass spectrometry as a routine laboratory procedure (Valbuena et al., 2016). Dose-adjusted C_0_ values were calculated by dividing the C_0_ by the total daily dose (nanogram per milliliter per milligram per day). Area under the time–concentration curve (AUC) was used as an estimate of the exposure to tacrolimus over time, since C_0_ was not available at every single postoperative day for every patient. Separate calculations were made for the first week (AUC_1–7days_), the second week (AUC_8–14days_), and for the whole follow-up period (AUC_1–16days_). AUC is reported as tacrolimus concentration divided by daily dose times days, i.e. (ng/mL)/ (mg/day) × days.

### Clinical Outcome Parameters

Clinical outcome measures for this study were AR, DGF, and renal function at discharge from the hospital. AR was defined as an acute deterioration in allograft function associated with specific pathologic changes in graft biopsies occurring during the first year after transplantation. DGF was defined as the need for dialysis within the entire 16 days post-transplantation for this study. Estimated glomerular filtration rate based on serum creatinine (eGFR) was used as measure for renal function at discharge from the hospital.

### Statistical Analysis

Statistical analyses were performed with R-3.5.0 (R Core Team, 2018), including additional packages coin_1.2-2, quantreg_5.36, and SNPassoc_1.9-2 (Koenker, 2004; Hothorn et al., 2006; González et al., 2007; Champely et al., 2018). Observed and expected allele and genotype frequencies within populations were compared using Hardy–Weinberg equilibrium calculations. Here, for the tri-allelic *ABCB12677G>T/A* variant, the HWTriExact function from the R-package HardyWeinberg_v.1.6.1 was used (Graffelman and Camarena, 2008; Graffelman, 2015). Linkage disequilibrium computation was performed using Haploview (Barrett et al., 2005).

AUC values were estimated from the available individual C_0_ levels using the trapezoid rule. The differences in quantitative variables (AUC, tacrolimus blood levels, and weight-adjusted daily tacrolimus doses) among individuals with different genotypes were investigated using Kruskal–Wallis tests, Wilcoxon–Mann–Whitney tests, or median regression as appropriate. The variants were considered in four different genetic models: codominant, dominant, recessive, and log additive. For the tri-allelic *ABCB1* 2677G>T,A variant, T and A allele were combined before applying these models.

These models were also applied for the multivariate logistic/linear regression analyses of genetic variants and their association with AR, DGF, or square root transformed eGFR. Here, human leukocyte antigen (HLA) mismatch (≥3 vs <3), panel reactive antibodies (>10% vs ≤10%), AB0 compatibility, (yes vs no), donor source (living vs. deceased), type of agent used for induction (basiliximab vs. thymoglobulin), previous transplantation (yes vs. no), and valgancyclovir therapy (yes vs. no) were considered as covariates. The association of AR, DGF, and eGFR with confounders was tested with Fisher tests; for the continuous variable eGFR, Wilcoxon rank sum tests were employed.

Post hoc power calculation was performed based on a two-sample t-test, the respective effect and samples sizes in our cohort, and a two-sided significance level of 5%. Weight-adjusted doses at day 10 were log-transformed in order to estimate coefficients of determination based on univariate linear models.

All statistical tests were two-sided, and significance level was set to 5%. Where indicated, P-values were adjusted for multiple testing according to Holm (1979).

## Results

### Genotyping Results and Genotype Frequencies

All genotype frequencies were in Hardy–Weinberg equilibrium. The two *CYP3A4* variants *1B and *22 were not linked (D’ = 0.19, r^2^ = 0.0). There were no homozygotes for the CYP3A4*22 allele, and 9.9% were heterozygous carriers. The minor allele frequency for CYP3A4*22 was 5%. Seventeen patients were heterozygous carriers for CYP3A5*1, and one patient carried two functional alleles (*1/*1). In total, 11 patients (8.7%) were classified as PM (CYP3A4*22 allele in combination with CYP3A5*3/*3), 93 patients (77.0%) as IM (CYP3A5*3/*3 plus CYP3A4*1/*1 or CYP3A4*22 carriers plus one CYP3A5*1 allele), and 17 patients (14.3%) as EM (CYP3A4*1/*1 carriers with at least one CYP3A5*1 allele). For the *ABCB1* variants, linkage was observed between 1236C>T and 2677G>T (D’ = 0.88, r^2^ = 0.72), 1236C>T and 3435C>T (D’ = 0.75, r^2^ = 0.45), as well as 2677G>T and 3435C>T (D’ = 0.95, r^2^ = 0.64). LD plots with D’ and r^2^ are shown in **Supplementary Figure 1**. Of our patients, 59.5% carried the T-T-T haplotype. Frequencies for all tested variants are given in **Table 2**.

**Table 2:** Genotype and allele frequencies of selected variants in candidate genes^#^.

Gene	Variant	Allele	%	Genotype	N (%)	HWE P value
*CYP3A4*		T	95.5	TT	110 (90.9)	1.0
	rs2740574 (*1B)	C	4.5	CT	11 (9.1)	
*CYP3A4*		G	95	GG	109 (90.1)	1.0
	rs35599367 (*22)	A	5	GA	12 (9.9)	
*CYP3A5*		T	7.9	TT	1 (0.8)	0.54
	rs776746 (*3)	C	92.1	TC	17 (14.1)	
				CC	103 (85.1)	
*ABCB1*		C	57.4	CC	43 (35.5)	0.27
	rs1128503 (1236C>T)	T	42.6	CT	53 (43.8)	
				TT	25 (20.7)	
*ABCB1*		G	57.85	GG	41 (33.9)	0.8^§^
	rs2032582 (2677G>T/A)^$^	T	40.5	GT/GA	58 (47.9)	
		A	1.65	TT/TA	22 (18.2)	
*ABCB1*		C	51.2	CC	35 (28.9)	0.27
	rs1045642 (3435C>T)	T	48.8	TC	54 (44.6)	
				TT	32 (26.4)	
*ABCB1* (1236, 2677, 3435)	TTT carriers 72 (59.5%)					
	non-carriers 49 (40.5%)					

HWE, Hardy–Weinberg equilibrium.

^#^Determined by TaqMan SNP genotyping assays.

^$^Only four patients were carriers of the A allele (3 patients: GA, 1 patient: TA).

^§^using HWTriExact function from the R-package HardyWeinberg.

### Effect of Genotypes on Tacrolimus Dose Requirements

**Figure 1:**
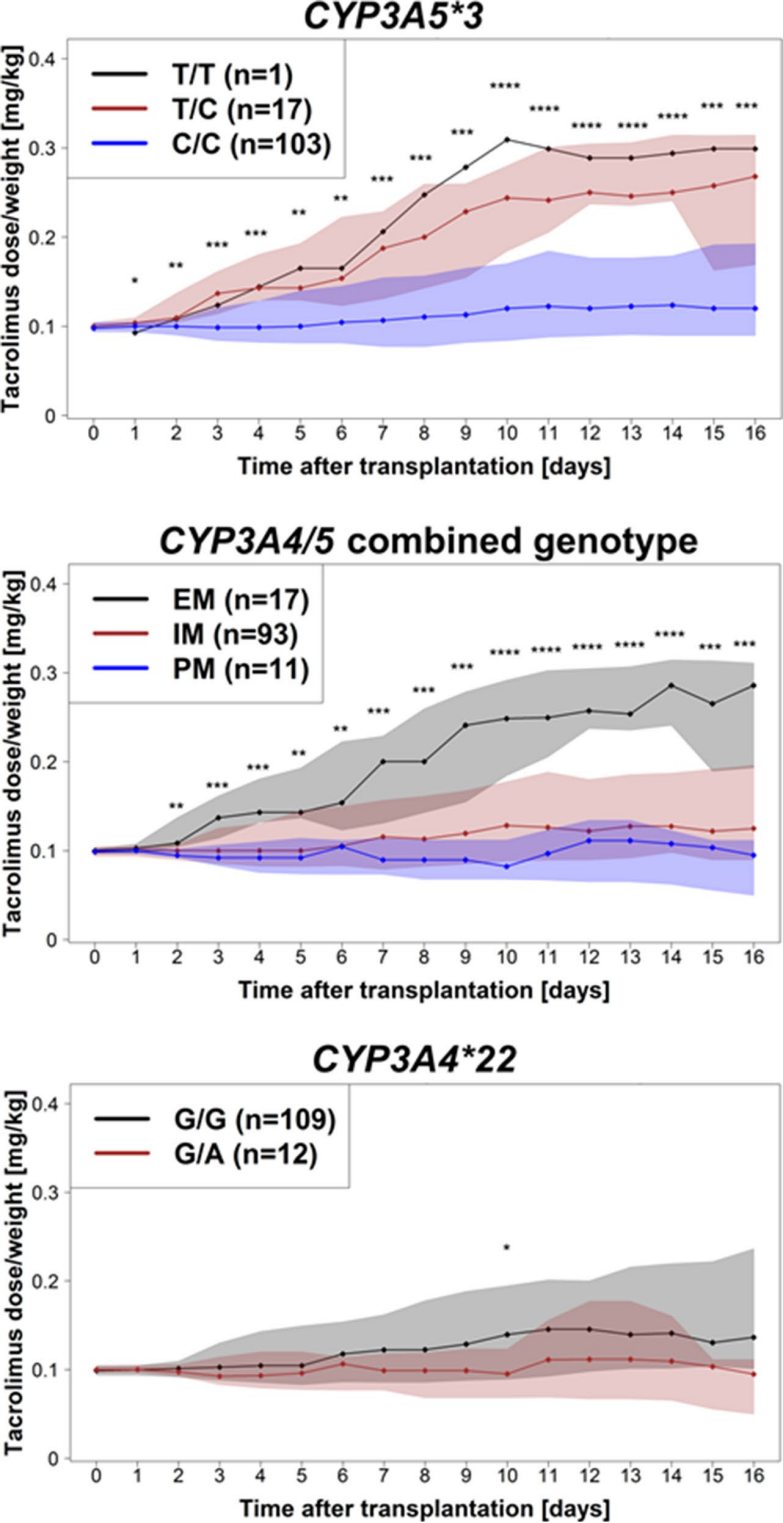
Tacrolimus dose requirement (mg/kg body weight/day) for the entire observation period of 16 days after transplantation for CYP3A5*3, *CYP3A4*/*CYP3A5* combined genotypes, and CYP3A4*22. Dots represent medians of tacrolimus dose/body weight at the different days; shaded areas are defined by 25 and 75% quantiles. Significance levels are shown as asterisk: *P < 0.05, **P < 0.01, ***P < 0.001, ****P < 0.0001.

As illustrated in **Figure 1**, already at day 1 after onset of therapy, a significantly higher dose requirement in CYP3A5 expressers (with at least one functional *CYP3A5* allele, n = 18) was observed compared with non-expressers (CYP3A5*3/*3; P = 0.046). This association remains significant for the total observation period of 16 days. These data are also supported by a significantly higher dose requirement (P = 0.004) for EM (n = 17) patients compared with both IM (n = 93) and PM (n = 11) subjects for the entire observation period. A trend of significance toward a decreased dose requirement in heterozygous CYP3A4*22 patients was evident after the first week of therapy, being statistically significant at day 10 (P = 0.03). A *post hoc* power calculation for CYP3A4*22 showed a power of 63.5%. Here, we used the data at day 10 after transplantation; a dose/weight showed a plateau after this time point. Moreover, based on univariate linear models with log-transformed weight-adjusted doses, estimated coefficients of determination were 2.7% for CYP3A4*1B, 4.8% for CYP3A4*22, 18.7% for CYP3A5*3, and 21.2% for the *CYP3A4/5* combined genotypes. In addition, carriers of CYP3A4*1B showed a significant higher tacrolimus dose requirement after day 12 of treatment (p = 0.026), keeping in mind that CYP3A4*1B and CYP3A5*3 are in strong linkage disequilibrium (D’ = 0.90, r^2^ = 0.45). None of the selected *ABCB1* variants showed significant effects on the tacrolimus dose requirement.

### Exposure to Tacrolimus

**Figure 2:**
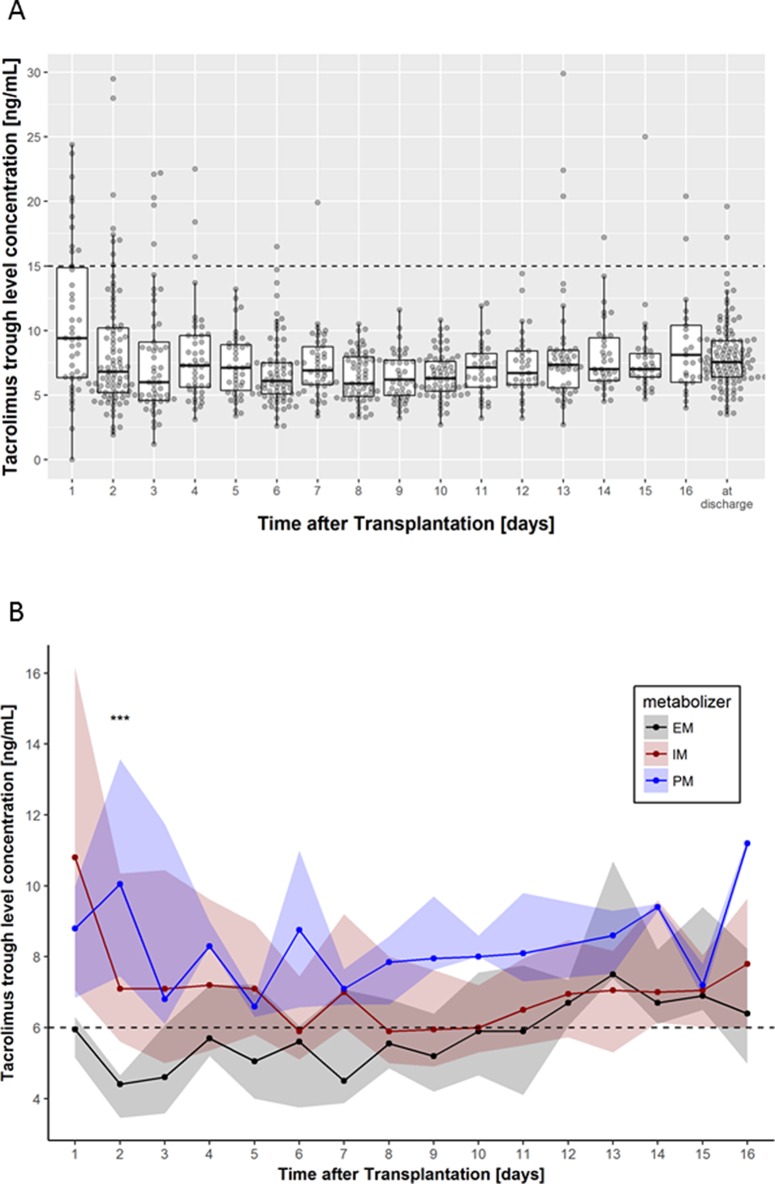
**(A)** Box-scatter plots per day of tacrolimus trough levels measured using liquid chromatography with tandem mass spectrometry. Thick lines represent median levels, boxes represent the interquartile range from 25 and 75% percentiles, and whiskers extend from the 25 or 75% percentile to the highest (or lowest, respectively) value not further than 1.5 times the interquartile range. The supra-therapeutic level of 15 ng/ml is shown using a horizontal dashed line. **(B)** Median tacrolimus trough levels according to *CYP3A4/5* combined genotype (EM, extensive metabolizer; IM, intermediate metabolizer, PM, poor metabolizer). Dots represent the medians per day, while shaded areas are defined by 25 and 75% quantiles. Levels below 6 ng/ml are considered to be subtherapeutic. Significance levels are shown as asterisk: ***P < 0.001.

An overview of the distribution of median tacrolimus levels for the entire observation period is shown in **Figure 2A**. Individuals carrying two nonfunctional alleles of CYP3A5*3/*3 had significantly increased AUCs calculated from the C_0_ measurements compared with CYP3A5 expressers even after consideration of multiple testing (log additive model: first week P = 9.60 × 10^-4^, second week P = 8.54 × 10^-4^, days 1–16 P = 3.34 × 10^-4^). In the same timeframe, significant increased AUCs were found in PM and IM compared with EM (log additive model: first week P = 0.002, second week P = 3.86 × 10^-5^, days 1–16 P = 1.54 × 10^-4^). Heterozygous carriers of the CYP3A4*22 variant showed significantly higher median AUCs from days 8–14 (log additive model: adjusted P = 0.016), while for the entire study period of 16 days, only a trend of significance was found (adjusted P = 0.176). Results for the tested *CYP3A4* and *CYP3A5* variants are shown in **Table 3**. For CYP3A4*1B and variants of *ABCB1* (individual variants as well as the T-T-T haplotype), no significant effects on the AUC of dose-adjusted tacrolimus concentrations were revealed. **Table 2** of the **Supplementary Material** summarizes the results for all tested variants and genetic models.

**Table 3:** Univariate analysis of AUC of dose-adjusted tacrolimus trough levels for *CYP3A4* and *CYP3A5* variants.

Gene	Variant	Genetic Model	AUC (ng/ml/mg/day * days) of dose-adjusted tacrolimus trough levels								
			Day 1-7			Day 8-14			Day 1-16		
			Median ± IQR	Effect size	P value	Median ± IQR	Effect size	P value	Median ± IQR	Effect size	P value
*CYP3A4*	*1B, T>C rs2740574	dominant: TC vs TT	3.8 ± 3.5 vs 5.8 ± 4.2	-1.6 (-3.4, -0.02)	0.046^§^	3.8 ± 3.9 vs 4.6 ± 3.4	-0.77(-2.3, 0.69)	0.249	9.3 ± 10 vs 12 ± 8.6	-3.2 (-6.8, 0.33)	0.071
		log additive: 0, 1, 2	5.5 ± 3.9	-1 (-2,0)	0.048^§^	4.5 ± 3.5	0 (-1, 1)	0.244	12 ± 8.9	-1 (-4, 1)	0.072
*CYP3A4*	*22, G>A rs35599367	dominant: GA vs GG	6.7 ± 3.4 vs 5.3 ± 4.0	1 (-0.65,2.9)	0.173	6.9 ± 4.1 vs 4.2 ± 3.5	2.4 (1.0, 4.1)	**0.002**	17 ± 10 vs 12 ± 8.9	4.3 (0.6, 9.1)	0.027^§^
		log additive: 0, 1, 2	5.5 ± 3.9	1 (-1,2)	0.17	4.5 ± 3.5	2 (1, 5)	**0.003**	12 ± 8.9	4 (0, 8)	0.029^§^
*CYP3A5*	*3, T>C rs776746	dominant TT + TC vs CC	2.9 ± 2 vs 6.0 ± 4.1	-2.3 (-3.6,-1.2)	**6 × 10^-5^**	2.5 ± 1.8 vs 4.9 ± 3.3	-1.9 (-3, -0.9)	**6.1 × 10^-5^**	6.7 ± 5.9 vs 13 ± 8.7	-5.2 (-7.9, -3)	**1.5 × 10^-5^**
		log additive: TT = 0, TC = 1, CC = 2	5.5 ± 3.9	-3 (-3,-2)	**1.2 × 10^-4^**	4.5 ± 3.5	-2 (-3, -2)	**1.2 × 10^-4^**	12 ± 8.9	-6 (-8, -4)	**4.8 × 10^-5^**
*CYP3A*	CYP3A4/5 combined genotypes ^†^	dominant: IM + PM vs EM	6 ± 4 vs 2.8 ± 1.7	2.4 (1.2,3.7)	**5.8 × 10^-5^**	4.9 ± 3.2 vs 2.4 ± 1.4	2 (1, 3.1)	**1.8 × 10^-5^**	13 ± 8.5 vs 6.7 ± 3.9	5.3 (3.1, 8.1)	**1.1 × 10^-5^**
		log additive: EM = 0, IM = 1, PM = 2	5.5 ± 3.9	2 (1.4,3.1)	**2.9 × 10^-4^**	4.5 ± 3.5	2.5 (1.7, 3.0)	**4.8 × 10^-6^**	12 ± 8.9	5.6 (4.3, 7.0)	**1.9 × 10^-5^**

95% CI, 95% confidence interval; IQR, interquartile range; AUC, area under the curve; P-values are unadjusted P-values. P-values in bold remain significant after adjustment for multiple comparison. Wilcoxon–Mann–Whitney test was used for single variants; the Kruskal–Wallis test or linear median regression analysis was used for CYP3A4/5 combined genotypes (^†^according to Elens et al., 2013, Lloberas et al., 2017).

### Supra-Therapeutic and Subtherapeutic Tacrolimus Levels

Thirty six of 754 measurements showed supra-therapeutic tacrolimus levels (defined as >15 ng/ml). Frequencies of supra-therapeutic measurements were not different (P = 0.36) between EM (3/111), IM (28/575), or PM (5/68). Of the 36 supra-therapeutic levels, 27 fell into the first 4 days of treatment, and 10 of them occurred on the first day (**Figure 2A**). In addition, five patients (1 EM and 3 IM) showed supra-therapeutic levels only after day 13.

Subtherapeutic tacrolimus levels (defined as <6 ng/ml) during the entire observation time were more common in EM patients (54%; P < 4.7 × 10^-9^) compared with IM (37%) or PM (9%). Moreover, the target C_0_ tacrolimus level of >6 ng/ml was achieved in EM patients not before day 12 of onset of therapy (**Figure 2B**).

### Impact of *CYP3A4/5* and *ABCB1* Variants on Clinical Outcome

AR in the first year after transplantation and DGF within the observation period of 16 days were observed in 18 patients (14.9%) and 30 patients (25%), respectively. eGFR at discharge from the hospital was >60 ml/min in 27 patients. Most patients achieved an eGFR between >30 and ≤59 ml/min (n = 64 patients). Seventeen patients retained a severely decreased renal function (>15 and ≤30 ml/min), while 12 patients had an eGFR below <15 ml/min at discharge.

No statistical significant effects of all tested genetic variants and related phenotypes (EM, IM, and PM) on AR, DGF, or renal function were found after correction for multiple comparisons (**Table 4**). However, AR was significantly associated with ≥3 HLA allelic mismatches (OR = 12.14, 95% CI 1.76, 525.21, P = 0.019 even after multiple testing). Donor source (deceased vs. living) was associated with a higher frequency of DGF (OR 7.15, 95% CI 2.23, 30.46, adjusted P = 0.0008) and a lower eGFR at discharge (mean 37.41 ml/min, SD ± 18.58 ml/min versus mean 53.75 ml/min, SD ± 18.58 ml/min, P = 0.0001) (**Table 5**). With regard to concomitant medication, valgancyclovir treatment did not impact on AR, DGF, or eGFR.

**Table 4:** Impact of selected CYP3A4/5 and ABCB1 variants on acute rejection, delayed graft function and estimated glomerular filtration rate at discharge (multivariate regression analysis).

Gene	Variant	Genetic Model	Acute Rejection		Delayed graft function		eGFR^#^	
			OR (95% CI)	P value	OR (95% CI)	P value	Effect (95% CI)	P value
*CYP3A4*	*1B, T>C rs2740574	dominant: TC vs TT	1.42 (0.21, 9.38)	0.72	2.39 (0.49, 11.55)	0.28	-0.29 (-1.26, 0.68)	0.56
		log additive: 0,1,2	1.42 (0.21, 9.38)	0.72	2.39 (0.49, 11.55)	0.28	-0.29 (-1.26, 0.68)	0.56
*CYP3A4*	*22, G>A rs35599367	dominant: GA vs GG	1.79 (0.24, 13.83)	0.58	0.89 (0.15, 5.23)	0.89	0.15 (-0.79, 1.1)	0.75
		log additive: 0,1,2	1.79 (0.24, 13.83)	0.58	0.89 (0.15, 5.23)	0.89	0.15 (-0.79, 1.1)	0.75
*CYP3A5*	*3, T>C rs776746	dominant: TT + TC vs CC	1.78 (0.43, 7.32)	0.43	2.07 (0.58, 7.38)	0.26	-0.73 (-1.50, 0.05)	0.068
		log additive: 0,1,2	1.76 (0.43, 7.18)	0.44	2.18 (0.69, 6.87)	0.18	-0.74 (-1.45, -0.04)	0.042^+^
*CYP3A*	CYP3A4/5 combined genotypes^†^	dominant: IM + PM vs EM	0.46 (0.11, 1.98)	0.31	0.47 (0.13, 1.69)	0.25	0.82 (0.02, 1.61)	0.046^+^
		log additive: EM = 0, IM = 1, PM = 2	0.82 (0.24, 2.76)	0.74	0.61 (0.22, 1.70)	0.34	0.45 (-0.12, 1.03)	0.12
*ABCB1*	1236C>T rs1128503	dominant: CT + TT vs CC	1.54 (0.41, 5.83)	0.52	1.44 (0.53, 3.90)	0.47	-0.03 (-0.63, 0.58)	0.94
		log additive: 0, 1, 2	1.22 (0.54, 2.75)	0.63	1.18 (0.62, 2.25)	0.61	0.00 (-0.39, 0.39)	1
*ABCB1*	2677G>T/A^##^ rs2032582	dominant: GT + TT vs GG	7.38 (1.07, 51.05)	0.016^+^	1.23 (0.43, 3.47)	0.70	-0.18 (-0.80, 0.44)	0.57
		log additive: 0, 1, 2	1.8 (0.75, 4.29)	0.18	0.97 (0.49, 1.94)	0.93	-0.13 (-0.53, 0.28)	0.54
*ABCB1*	3435C>T rs1045642	dominant: CT + TT vs CC	5.06 (0.84, 30.52)	0.043^+^	1.95 (0.63, 6.02)	0.24	-0.38 (-1.01, 0.25)	0.24
		log additive: 0, 1, 2	2.14 (0.91, 5.04)	0.071	0.90 (0.46, 1.74)	0.75	-0.12 (-0.51, 0.26)	0.53
*ABCB1*	TTT haplotype (1236,2677,3435)	dominant: carriers vs non-carriers	2.2 (0.6, 8.06)	0.22	1.62 (0.61, 4.29)	0.33	-0.27 (-0.85, 0.32)	0.37
		log additive: 0, 1, 2	2.2 (0.6, 8.06)	0.22	1.62 (0.61, 4.29)	0.33	-0.27 (-0.85, 0.32)	0.37

eGFR, estimated glomerular filtration rate at discharge, OR, odds ratio, CI, confidence interval, Multivariate analysis includes HLA mismatch <3 alleles vs. ≥3 alleles, % panel reactive antibodies: ≤10% (low risk) vs >10% (high risk), AB0 compatibility: yes vs. no, previous transplantation: yes vs. no, living vs. deceased donor, basiliximab vs. thymoglobulin, valgancyclovir yes vs. no.

^#^ Square root transformed eGFR value.

^##^ The A allele was combined with T allele previous to statistical analysis, i.e. GA carriers were treated as GT carriers and TA carriers as TT carriers.

^+^ Not significant after correction for multiple comparisons.

^†^ According to Elens et al., 2013, Lloberas et al, 2017.

**Table 5:** Influence of clinical confounding factors^$^.

	Odds ratio^§^	95 % CI	P value	Adjusted P value^#^
**Acute rejection (AR)**				
HLA mismatch ≥ 3 alleles vs HLA mismatch <3 alleles	12.14	1.76, 525.21	0.0027	0.019
**Delayed graft function (DGF)**				
Graft from deceased vs living donor	7.15	2.23, 30.46	0.0001	0.0008
	Estimate*	95 % CI	P value	Adjusted P value^#^
**eGFR at discharge**				
Graft from deceased vs living donor	-17.00	-24.00, -9.00	0.00001	0.0001

CI, confidence interval; HLA, human leukocyte antigen; eGFR, estimated glomerular filtration rate.

^$^Table shows only confounders which were significantly associated with one of the outcome measures AR, DGF, or eGFR. Confounders tested: HLA alleles, vPRA, virtual panel reactive antibodies, donor source, previous transplantation, valgancyclovir therapy, AB0 compatibility, type of induction therapy.

*Difference in location (Wilcoxon rank sum test) from eGFR at discharge (mL/min).

^§^Fisher test.

^#^Adjusted for multiple comparisons according to Holm’s procedure.

## Discussion

This study aimed to elucidate the impact of CYP3A*22 and *CYP3A5* and selected functional relevant *ABCB1* variants on pharmacokinetics and outcome parameters in clinical practice in a well-defined single center cohort of renal transplant patients treated with tacrolimus.

### Tacrolimus Dose Requirements

An important aspect to this study was to investigate the effect of the variants on the tacrolimus dose. To minimize rejection and toxicity after transplantation, the target tacrolimus C_0_ level at our center is 6–8 ng/ml during the first 3 months, which is in agreement with international recommendations (Kasiske et al., 2009). Already at day 1, CYP3A5 expressers (n = 18) had significantly (P = 0.046) higher dose requirements than CYP3A5 non-expressers (CYP3A5*3/*3). For the *CYP3A4/5* combined genotypes, EM (n = 17) showed also a significantly higher dose requirement than both IM (n = 93) or PM (n = 11) at onset of therapy. The IM and PM groups did not show a significant difference in the daily doses, in contrast to Gijsen et al. (2013). In line with other studies (Elens et al., 2011a; Elens et al., 2013; Gijsen et al., 2013; Pallet et al., 2015), tacrolimus dosing for the entire observation time was lower in patients who were heterozygous carriers of CYP3A4*22. However, statistically significant differences between CYP3A4*1/*22 and CYP3A4*1/*22 were only observed at day 10 keeping in mind that the number of variant carriers for CYP3A4*22 is low in our study cohort. The *CYP3A4/5* combined genotypes explain a greater part of variability in dose requirement (21.2%) compared with the isolated contribution of CYP3A4*22 (4.8%) corroborating previous data about the relevance of *CYP3A5* for tacrolimus dosing.

### CYP3A4*22 and Tacrolimus Pharmacokinetics

The wide range of tacrolimus C_0_ levels observed in patients treated with the same dose of tacrolimus can be explained in part by the genetic variation in *CYP3A5*. Yet, although CYP3A4*22 contributes to a lower rate to tacrolimus metabolism, CYP3A4*22 related higher C_0_ tacrolimus levels have been reported in some cohorts (Elens et al., 2011a). For instance, the impact of CYP3A4*22 was described by Scheibner et al. (2018), who reported a median 342% increase of dose-adjusted tacrolimus C_0_ levels in transplant patients homozygous for CYP3A4*22 and CYP3A5*3 allele, compared with controls.

In our cohort, the minor allele frequency for the CYP3A4*22 allele was 5%, i.e., 12 of 121 patients carried the CYP3A4*22 allele heterozygously. This frequency is in line with the allele frequency of 4.4% in non-Finnish Europeans as reported by the Genome Aggregation Database (Lek et al., 2016) and other research groups (van Schaik et al., 2002; Elens et al., 2013). Classification into extensive (EM, 14%), intermediate (IM, 77%), and PM (9%) revealed a similar distribution as reported by Lloberas et al. (2017).

The exposure to tacrolimus (AUC of dose-adjusted levels) in our study was significantly higher (P = 0.016) in heterozygous CYP3A4*22 carriers compared with CYP3A4*1/*1 (days 8 to 14). This observation holds true for the entire observation period up to 16 days with a trend of significance after correction for multiple testing. Several studies reported a significantly reduced tacrolimus clearance (with a higher C_0_) in heterozygous patients for CYP3A4*1/*22 in the early period after renal transplantation (Elens et al., 2011a; Pallet et al., 2015; Lloberas et al., 2017). In contrast, other studies did not find clinically significant effects for this association (Tavira et al., 2011; Santoro et al., 2013; Moes et al., 2014). One explanation for this observation is that different time frames were selected to assess tacrolimus clearance, exemplarily demonstrated by the studies of Elens et al. (2011a) and Lloberas et al. (2017) with a time frame up to 1 year after transplantation.

### *CYP3A5* and *CYP3A4/5* Combined Genotype and Tacrolimus Pharmacokinetics

Throughout the first 16 days post-transplantation, CYP3A5 non-expressers (CYP3A5*3/*3) showed significantly higher dose-adjusted tacrolimus AUC than expressers (CYP3A5*1/*3 or CYP3A5*1/*1). This resembles data from a meta-analysis by Rojas et al. (2015). Regarding the *CYP3A4/5* combined genotypes, both the PM or IM cluster have significantly higher dose-adjusted tacrolimus AUCs in the first 16 days post-transplantation than EM. AUCs show a huge interindividual variability in IM and PM and do not differ significantly between these groups. Assessment of the *CYP3A4/5* combined genotypes regarding AUC was not superior compared with the monogenic *CYP3A5* analysis.

### Supra- and Subtherapeutic Levels

Few studies report on an increased risk for supra-therapeutic tacrolimus concentrations (C_0_ > 15ng/ml) in heterozygous carriers of CYP3A4*22 (Pallet et al., 2015) as well as in homozygous-variant patients (Lloberas et al., 2017). In the former study, an initial dose of 0.2 mg/kg body weight has been used, which is twice the daily dose used in our protocol. The latter study aimed for a higher target C_0_ in the first week of transplantation. Most probably due to our lower dosing regimen (0.1 mg/kg body weight), supra-therapeutic tacrolimus levels were found only on 36 of 754 occasions, with a majority occurring in the first 4 days of tacrolimus therapy (**Figure 2A**). Supra-therapeutic levels in our cohort were not associated with the *CYP3A4/5* combined genotype, which may also be explained by lower dosing of tacrolimus. Subtherapeutic levels (<6 ng/ml) may be a concern in CYP3A5 expressers or EM patients due to an enhanced metabolism toward inactive metabolites (Lloberas et al., 2017). In our cohort, measurements of <6 ng/ml were in fact most common in EM patients. In consequence, during the first 11 days of therapy, the median C_0_ was below the target value of 6 ng/ml in EM.

### Clinical Outcome

Generally, highest incidence of AR is reported within the first year after transplantation (The Scientific Registry of Transplant Recipients/Organ Procurement and Transplantation Network, 2012). Although genetic variation in drug metabolism has been confirmed to lead to different tacrolimus blood levels, a meta-analysis concluded that tacrolimus C_0_ does not predict the risk of AR after renal transplantation (Bouamar et al., 2013).

These data are supported by our study indicating that variation in *CYP3A* and/or *ABCB1* did not impact on AR, DGF, or renal function (eGFR) on discharge, when controlling for confounders like HLA mismatch, % panel reactive antibodies, AB0 compatibility, valgancyclovir use, donor source, number of transplant, and type of induction therapy. CYP3A4*22 has been associated with DGF, but only with cyclosporine (Elens et al., 2012) and not for tacrolimus use. HLA mismatches are generally recognized as a major barrier to long-term engraftment and supported by our data. AR occurring within 1 year of transplantation was significantly associated with ≥3 HLA allelic mismatches. Moreover, DGF and a reduced eGFR at discharge were significantly more frequent in recipients with a deceased donor graft. These observations corroborate previous data (Perico et al., 2004; Ghods et al., 2007; Siedlecki et al., 2011) and underline the major impact of transplantation relevant factors like the HLA mismatches and the donor source of the transplant for clinical outcome variables.

### Limitations

Our study has some limitations. It is a retrospective analysis study. The number of patients in our single-center study was relatively small (n = 121), which may explain why we did not observe significant effects of the tested genotypes on the clinical endpoints. We only performed genetic analysis using DNA from recipients but not from donors, which may explain missing genetic associations observed by others, e.g., *ABCB1* variants and DGF (Woillard et al., 2010; Hauser et al., 2012). Although all our transplant patients received the same standard therapeutic regimen and we explicitly considered concomitant valgancyclovir therapy as a confounding factor in our multivariate analysis, additional medications given to transplant recipients may impact tacrolimus pharmacokinetics with consequences on outcome variables.

### Conclusion

Our results confirm previous data indicating that *CYP3A* variants explain interindividual variability of tacrolimus pharmacokinetics but do not impact on clinical outcome variables like AR, DLF, or eGFR. Notably, CYP3A4*22 isolated or in combination with *CYP3A5* genotypes did not decisively improve the well-established value of *CYP3A5* pharmacogenetic testing only to predict tacrolimus dosing. *ABCB1* did neither significantly impact on tacrolimus pharmacokinetics nor on clinical outcome parameters.

## Data Availability

The raw data supporting the conclusions of this manuscript will be made available by the authors, without undue reservation, to any qualified researcher.

## Ethics Statement

All subjects have given their written informed consent. The study was approved by the ethics committee of University Hospital, Tuebingen, Germany (616/2013BO2).

## Author Contributions

EA-K was responsible for acquisition of all source data and contributed to the writing of the manuscript. SW performed or supervised statistical analyses and critically revised the manuscript. RT performed statistical analyses. ES was responsible for genotyping. CO recruited the patients and provided the clinical data. EW, MSc, and MSh were responsible for funding, conception of this study, interpretation of results, and critical revision of the manuscript. SJ performed statistical analysis and wrote the manuscript.

## Funding

This study was supported in part by the Robert Bosch Stiftung Stuttgart, Germany and in part by the Horizon 2020-PHC-2015 grant U-PGx 668353.

## Conflict of Interest Statement

The authors declare that the research was conducted in the absence of any commercial or financial relationships that could be construed as a potential conflict of interest.
