# Supplementary material for: The Impact of CYP3A4*22 on Tacrolimus Pharmacokinetics and Outcome in Clinical Practice at a Single Kidney Transplant Center
Source: Front Genet. 2019 Sep 26;10:871. doi: 10.3389/fgene.2019.00871 (PMC6775237; doi:10.3389/fgene.2019.00871)

Supplementary Table 1 – Univariate analysis of AUC of dose-adjusted tacrolimus trough levels

| **Gene** | **Variant** | **Genetic Model** | **AUC (ng/mL/mg/day*days) of dose-adjusted tacrolimus trough levels** | | | | | | | | |
| --- | --- | --- | --- | --- | --- | --- | --- | --- | --- | --- | --- |
|  |  |  | **Day 1-7** | | | **Day 8-14** | | | **Day 1-16** | | |
|  |  |  | **Median**  ± **IQR** | **Effect size** | **P value** | **Median**  ± **IQR** | **Effect size** | **P value** | **Median**  ± **IQR** | **Effect size** | **P value** |
| *CYP3A4* | *1B, T>C  rs2740574 | codominant:  TC vs TT | 3.8 ± 3.5 vs  5.8 ± 4.2 | -1.6  (-3.4, -0.02) | 0.046^§^ | 3.8 ± 3.9 vs  4.6 ± 3.4 | -0.77 (-2.3,0.69) | 0.249 | 9.3 ± 10 vs  12 ± 8.6 | -3.2 (-6.8,0.33) | 0.071 |
|  |  | dominant:  TC vs TT | 3.8 ± 3.5 vs  5.8 ± 4.2 | -1.6  (-3.4, -0.02) | 0.046^§^ | 3.8 ± 3.9 vs  4.6 ± 3.4 | -0.77 (-2.3,0.69) | 0.249 | 9.3 ± 10 vs  12 ± 8.6 | -3.2 (-6.8,0.33) | 0.071 |
|  |  | log additive: 0,1,2 | 5.5 ± 3.9 | -1 (-2,0) | 0.048^§^ | 4.5 ± 3.5 | 0 (-1,1) | 0.244 | 12 ± 8.9 | -1 (-4,1) | 0.072 |
| *CYP3A4* | *22, G>A  rs35599367 | codominant  GA vs GG | 6.7 ± 3.4 vs  5.3 ± 4.0 | 1  (-0.65,2.9) | 0.173 | 6.9 ± 4.1 vs  4.2 ± 3.5 | 2.4 (1.0,4.1) | **0.002** | 17 ± 10 vs  12 ± 8.9 | 4.3 (0.6,9.1) | 0.027^§^ |
|  |  | dominant:  GA vs GG | 6.7 ± 3.4 vs  5.3 ± 4.0 | 1  (-0.65,2.9) | 0.173 | 6.9 ± 4.1 vs  4.2 ± 3.5 | 2.4 (1.0,4.1) | **0.002** | 17 ± 10 vs  12 ± 8.9 | 4.3 (0.6,9.1) | 0.027^§^ |
|  |  | log additive: 0,1,2 | 5.5 ± 3.9 | 1 (-1,2) | 0.17 | 4.5 ± 3.5 | 2 (1,5) | **0.003** | 12 ± 8.9 | 4 (0,8) | 0.029^§^ |
| *CYP3A5* | *3, T>C  rs776746 | codominant:  TT vs TC vs CC | TC vs CC:  3.1 ± 2.1 vs  6 ± 4.1  TT vs CC:  1.7 ± 0 vs  6 ± 4.1 | TC vs CC:  -2.2 (-3.6,-1.1)  TT vs CC:  -4.2 (-22,-0.81) | **3.9 x 10^-4^** | TC vs CC:  2.6 ± 1.8 vs  4,9 ± 3.3  TT vs CC:  1.5 ± 0 vs  4,9 ± 3.3 | TC vs CC:  -1.8 (-2.9,-0.79)  TT vs CC:  -3.4 (-9.2,-045) | **3.9 x 10^-4^** | TC vs CC:  6.7 ± 5.9 vs  13 ± 8.7  TT vs CC:  3.9 ± 0 vs  13 ± 8.7 | TC vs CC:  -5 (-7.7,-2.6)  TT vs CC:  -9.1 (-35,-2.2) | **1.4 x 10^-4^** |
|  |  | dominant:  TT + TC vs CC | 2.9 ± 2 vs  6.0 ± 4.1 | -2.3 (-3.6,-1.2) | **6 x 10^-5^** | 2.5 ± 1.8 vs  4.9 ± 3.3 | -1.9 (-3,-0.9) | **6.1 x 10^-5^** | 6.7 ± 5.9 vs  13 ± 8.7 | -5.2 (-7.9,-3) | **1.5 x 10^-5^** |
|  |  | recessive:  TT vs CC + TC | 1.7 ± 0 vs  5.5 ± 3.9 | -3.8 (-22,-0.32) | 0.017^§^ | 1.5 ± 0 vs  4.6 ± 3.6 | -3.1 (-9.2,-0.17) | 0.017^§^ | 3.9 ± 0 vs  12 ± 8.8 | -8.4 (-35,-1.4) | 0.017^§^ |
|  |  | log additive: TT=0, TC=1, CC=2 | 5.5 ± 3.9 | -3 (-3,-2) | **1.2 x 10^-4^** | 4.5 ± 3.5 | -2 (-3,-2) | **1.2 x 10^-4^** | 12 ± 8.9 | -6 (-8,-4) | **4.8 x 10^-5^** |
| *CYP3A* | *CYP3A4/5* combined genotypes † | codominant:  PM vs IM vs EM | EM: 2.8 ± 1.7  IM: 5.8 ± 4.1  PM: 6.7 ± 3.2 | IM vs EM:  2.3 (1.2,3.6)  PM vs EM:  3.6 (1.2,5.0) | **3.4 x 10^-4^** | EM: 2.4 ± 1.4  IM: 4.6 ± 3.2  PM: 7.5 ± 4.4 | IM vs EM:  1.8 (0.87,2.9)  PM vs EM:  4.4 (2.0,6.7) | **1 x 10^-5^** | EM: 6.7 ± 3.9  IM: 12 ± 8.2  PM: 18 ± 9.4 | IM vs EM:  5.1 (2.8,7.6)  PM vs EM:  10 (4.3,15) | **3.2 x 10^-5^** |
|  |  | dominant:  IM + PM vs  EM | 6 ± 4 vs  2.8 ± 1.7 | 2.4 (1.2,3.7) | **5.8 x 10^-5^** | 4.9 ± 3.2 vs  2.4 ± 1.4 | 2 (1,3.1) | **1.8 x 10^-5^** | 13 ± 8.5 vs  6.7 ± 3.9 | 5.3 (3.1,8.1) | **1.1 x 10^-5^** |
|  |  | recessive:  PM vs EM + IM | 6.7 ± 3.2 vs  5.3 ± 4 | 1.3 (-0.48,3.3) | 0.125 | 7.5 ± 4.4 vs  4.3 ± 3.5 | 2.6 (1,4.5) | 0.193 | 18 ± 9.4 vs  12 ± 8.8 | 5.2 (1.1,10) | 0.018^§^ |
|  |  | log additive: EM=0, IM=1, PM=2 | 5.5 ± 3.9 | 2 (1.4,3.1) | **2.9 x 10^-4^** | 4.5 ± 3.5 | 2.5 (1.7,3.0) | **4.8 x 10^-6^** | 12 ± 8.9 | 5.6 (4.3,7.0) | **1.9 x 10^-5^** |

Supplementary Table 1 – CONTINUED

| **Gene** | **Variant** | **Genetic Model** | **AUC (ng/mL/mg/day*days) of dose-adjusted tacrolimus trough levels** | | | | | | | | |
| --- | --- | --- | --- | --- | --- | --- | --- | --- | --- | --- | --- |
|  |  |  | **Day 1-7** | | | **Day 8-14** | | | **Day 1-16** | | |
|  |  |  | **Median**  ± **IQR** | **Effect size** | **P value** | **Median**  ± **IQR** | **Effect size** | **P value** | **Median**  ± **IQR** | **Effect size** | **P value** |
| *ABCB1* | 1236C>T  rs1128503 | codominant:  TT vs CT vs CC | CT vs CC:  5.3 ± 5.4 vs  5.2 ± 3.1  TT vs CC:  6.1 ± 2.7 vs  5.2 ± 3.1 | CT vs CC:  0.33 (-0.8,1.5)  TT vs CC:  0.76 (-0.46,2) | 0.466 | CT vs CC:  4.2 ± 3.5 vs  4.6 ± 3.6  TT vs CC:  5 ± 2.7 vs  4.6 ± 3.6 | CT vs CC:  -0.22 (-1.1,0.78)  TT vs CC:  0.39 (-0.75,1.5) | 0.476 | CT vs CC:  12 ± 10 vs  12 ± 7.8  TT vs CC:  13 ± 6.6 vs  12 ± 7.8 | CT vs CC:  0.35 (-2.2,2.9)  TT vs CC:  1.4 (-1.3,4) | 0.584 |
|  |  | dominant:  CT + TT vs CC | 5.9 ± 4.3 vs  5.2 ± 3.1 | 0.48 (-0.52,1.5) | 0.317 | 4.5 ± 3.5 vs  4.6 ± 3.6 | 0.0014 (-0.87,0.87) | 0.997 | 12 ± 8.7 vs  12 ± 7.8 | 0.68 (-1.6,2.9) | 0.505 |
|  |  | recessive:  TT vs CC + CT | 6.1 ± 2.7 vs  5.3 ± 4.2 | 0.59  (-0.58,1.8) | 0.300 | 5.0 ± 2.7 vs  4.3 ± 3.7 | 0.47 (-0.51,1.4) | 0.262 | 13 ± 6.6 vs  12 ± 9.4 | 1.2 (-1.3,3.6) | 0.328 |
|  |  | log additive: 0,1,2 | 5.5 ± 3.9 | 0 (0,1) | 0.218 | 4.5 ± 3.5 | 0 (-1,1) | 0.53 | 12 ± 8.9 | 0 (-1,2) | 0.326 |
| *ABCB1* | 2677G>T/A^#^  rs2032582 | codominant:  TT vs GT vs GG | GT vs GG:  5.5 ± 4.5 vs  5.2 ± 3.4  TT vs GG:  6 ± 2.8 vs  5.2 ± 3.4 | GT vs GG:  0.32 (-0.79,1.5)  TT vs GG:  0.47 (-0.87,1.7) | 0.722 | GT vs GG:  3.7 ± 3.1 vs  4.92 ± 4  TT vs GG:  5 ± 2.3 vs  4.9 ± 4 | GT vs GG:  -0.55 (-1.7,0.39)  TT vs GG:  0.24 (-1,1.4) | 0.159 | GT vs GG:  11 ± 8.8 vs  12 ± 9.4  TT vs GG:  13 ± 6.8 vs  12 ± 9.4 | GT vs GG:  -0.23 (-2.9,2.2)  TT vs GG:  0.85 (-2.1,3.8) | 0.619 |
|  |  | cominant:  GT + TT vs GG | 5.5 ± 4 vs  5.2± 3.4 | 0.35 (-0.68,1.4) | 0.46 | 4.2 ± 3.4 vs  4.9 ± 4 | -0,29 (-1.3,0.57) | 0.498 | 12 ± 8.4 vs  12 ± 9.4 | 0.1 (-2.3,2.3) | 0.925 |
|  |  | recessive:  TT vs GG + GT | 6.0 ± 2.8 vs  5.4 ± 4.3 | 0.28 (-1.0,1.4) | 0.588 | 5.0 ± 2.3  4.2 ± 3.7 | 0.74 (-0.33,1.7) | 0.149 | 13 ± 6.8 vs  12 ± 9.3 | 1.3 (-1.5,3.7) | 0.347 |
|  |  | log additive: 0,1,2 | 5.5 ± 3.9 | 0 (-1,1) | 0.423 | 4.5 ± 3.5 | 0 (-1,1) | 0.736 | 12 ± 8.9 | 0 (-2,2) | 0.558 |
| *ABCB1* | 3435C>T  rs1045642 | codominant:  TT vs CT vs CC | CT vs CC:  4.8 ± 4.9 vs  6 ± 3.2  TT vs CC:  5.5 ± 2.9 vs  6 ± 3.2 | CT vs CC:  -0.5 (1.8,0.86)  TT vs CC:  -0.24 (-1.6,0.94) | 0.732 | CT vs CC:  3.6 ± 2.9 vs  5.2 ± 4.2  TT vs CC:  4.4 ± 2.8 vs  5.2 ± 4.2 | CT vs CC:  -1 (-2.2,0.092)  TT vs CC:  -0.29 (-1.6,0.91) | 0.121 | CT vs CC:  11 ± 9.4 vs  14 ± 7.6  TT vs CC:  12 ± 7.4 vs  14 ± 7.6 | CT vs CC:  -1.9 (-4.4,0.85)  TT vs CC:  -0.92 (-3.4,2) | 0.361 |
|  |  | dominant:  CT + TT vs CC | 5.3 ± 4.1 vs  6 ± 3.2 | -0.4 (-1.5,0.71) | 0.512 | 4.1 ± 3.3 vs  5.2 ± 4.2 | -0.72 (-1.8,0.24) | 0.146 | 12 ± 8.8 vs  14 ± 7.6 | -1.4 (-3.7,0.96) | 0.267 |
|  |  | recessive:  TT vs CC + CT | 5.5 ± 2.9 vs  5.5 ± 4.5 | 0.091 (-1.1,1.1) | 0.890 | 4.4 ± 2.8 vs  4.5 ± 3.6 | 0.36 (-0.63,1.2) | 0.450 | 12 ± 7.4 vs  12 ± 9.3 | 0.47 (-1.9,2.6) | 0.703 |
|  |  | log additive: 0,1,2 | 5.5 ± 3.9 | 0 (-1,1) | 0.75 | 4.5 ± 3.5 | 0 (-1,0) | 0.662 | 12 ± 8.9 | -1 (-2,1) | 0.651 |
| *ABCB1* | TTT haplotype (1236,2677,3435) | codominant: carriers  vs non-carriers | 5.5 ± 4.0 vs  5.5 ± 3.8 | 0.15 (-0.85,1.1) | 0.758 | 4.4 ± 3.3 vs  4.6 ± 4.0 | -0.081 (-0.96,0.74) | 0.852 | 12 ± 8.4 vs  12 ± 9.1 | 0.2 (-3.0,2) | 0.848 |
|  |  | dominant: carriers  vs non-carriers | 5.5 ± 4.0 vs  5.5 ± 3.8 | 0.15 (-0.85,1.1) | 0.758 | 4.4 ± 3.3 vs  4.6 ± 4.0 | -0.081 (-0.96,0.74) | 0.852 | 12 ± 8.4 vs  12 ± 9.1 | 0.2 (-3.0,2) | 0.848 |
|  |  | log additive: 0,1,2 | 5.5 ± 3.9 | 0 (-1,1) | 0.754 | 4.5 ± 3.5 | 0 (-1,1) | 0.85 | 12 ± 8.9 | 0 (-3,2) | 0.845 |

95% CI = 95% Confidence Interval; IQR = inter-quartile range; AUC = area under the curve; P-values are unadjusted P-values. P-values in bold remain significant after adjustment for multiple comparison. Wilcoxon-Mann-Whitney-Test was used for single variants and ABCB1 haplotype; the Kruskal-Wallis Test or linear median regression analysis were used for *CYP3A4/5* combined genotypes († according to Elens et al. 2013, Lloberas et al 2017).

# the A allele was combined with T allele previous to statistical analysis, i.e. GA carriers were treated as GT carriers and TA carriers as TT carriers

§ not significant after adjustment for multiple comparison (Holm correction)

Supplementary Table 2 − Multivariate logistic regression for acute rejection and delayed graft function and multivariate linear regression for GFR in carriers of the variants tested

| **Gene** | **Variant** | **Genetic Model** | **Acute Rejection** | | **Delayed graft function** | | **eGFR^#^** | |
| --- | --- | --- | --- | --- | --- | --- | --- | --- |
|  |  |  | **OR (95% CI)** | **P value** | **OR (95% CI)** | **P value** | **Effect**  **(95% CI)** | **P value** |
| *CYP3A4* | *1B, T>C  rs2740574 | codominant  TC vs TT | 1.42 (0.21,9.38) | 0.72 | 2.39 (0.49,11.55) | 0.28 | -0.29 (-1.26,0.68) | 0.56 |
|  |  | dominant  TC vs TT | 1.42 (0.21,9.38) | 0.72 | 2.39 (0.49,11.55) | 0.28 | -0.29 (-1.26,0.68) | 0.56 |
|  |  | log additive: 0,1,2 | 1.42 (0.21,9.38) | 0.72 | 2.39 (0.49,11.55) | 0.28 | -0.29 (-1.26,0.68) | 0.56 |
| *CYP3A4* | *22, G>A  rs35599367 | codominant  GA vs GG | 1.79 (0.24,13.93) | 0.58 | 0.89 (0.15,5.23) | 0.89 | 0.15 (-0.79,1.1) | 0.75 |
|  |  | dominant  GA vs GG | 1.79 (0.24,13.83) | 0.58 | 0.89 (0.15,5.23) | 0.89 | 0.15 (-0.79,1.1) | 0.75 |
|  |  | log additive: 0,1,2 | 1.79 (0.24,13.83) | 0.58 | 0.89 (0.15,5.23) | 0.89 | 0.15 (-0.79,1.1) | 0.75 |
| *CYP3A5* | *3, T>C  rs776746 | codominant:  TT vs TC vs CC | TC vs CC:  1.78 (0.43,7.34)  TT vs CC:  0 | 0.73 | TC vs CC:  1.67 (0.44,6.35)  TT vs CC:  -^§^ | 0.26 | TC vs CC:  -0.62 (-1.41,0.18)  TT vs CC:  -2.42 (-5.42,5.48) | 0.10 |
|  |  | dominant  TT + TC vs CC | 1.78 (0.43,7.32) | 0.43 | 2.07 (0.58,7.38) | 0.26 | -0.73 (1.50,0.05) | 0.068 |
|  |  | recessive:  TT vs CC + TC | -^§^ | -^§^ | -^§^ | -^§^ | -^§^ | -^§^ |
|  |  | log additive: 0,1,2 | 1.76 (0.43,7.18) | 0.44 | 2.18 (0.69,8.87) | 0.18 | -0.74 (-1.45,0.04) | 0.042^+^ |
| *CYP3A* | *CYP3A4/5*  combined genotypes † | codominant:  PM vs IM vs EM | IM vs EM:  0.40 (0.09,1.8)  PM vs EM:  1.23 (0.12,12.13) | 0.35 | IM vs EM:  0.47 (0.13,1.7)  PM vs EM:  0.48 (0.06,3.87) | 0.52 | IM vs EM:  0.82 (0.02,1.63)  PM vs EM:  0.77 (-0.40,1.94) | 0.14 |
|  |  | dominant:  IM + PM vs EM | 0.46 (0.11,1.98) | 0.31 | 0.47 (0.13,1.69) | 0.25 | 0.82 (0.02,1.61) | 0.046^+^ |
|  |  | recessive:  PM vs EM + IM | 2.49 (0.33,18.52) | 0.39 | 0.91 (0.15-5.41) | 0.92 | 0.09 (-0.88,1.06) | 0.86 |
|  |  | log additive: EM=0, IM=1, PM=2 | 0.82 (0.24,2.76) | 0.74 | 0.61 (0.22,1.70) | 0.34 | 0.45 (-0.2,1.03) | 0.12 |

Supplementary Table 2 − CONTINUED

| **Gene** | **Variant** | **Genetic Model** | **Acute Rejection** | | **Delayed graft function** | | **eGFR^#^** | |
| --- | --- | --- | --- | --- | --- | --- | --- | --- |
|  |  |  | **OR (95% CI)** | **P value** | **OR (95% CI)** | **P value** | **Effect**  **(95% CI)** | **P value** |
| *ABCB1* | 1236C>T  rs1128503 | codominant:  TT vs CT vs CC | CT vs CC:  1.57 (0.38,6.47)  TT vs CC:  1.46 (0.27,7.82) | 0.81 | CT vs CC:  1.49 (0.52,4.28)  TT vs CC:  1.33 (0.36,4.97) | 0.76 | CT vs CC:  -0.04 (-0.69,0.61)  TT vs CC:  0.01 (-0.78,0.80) | 0.99 |
|  |  | dominant:  CT + TT vs CC | 1.54 (0.41,5.83) | 0.52 | 1.44 (0.53,3.90) | 0.47 | -0.03 (-0.63,0.58) | 0.94 |
|  |  | recessive:  TT vs CC + CT | 1.1 (0.27,4.42) | 0.9 | 1.05 (0.33,3.29) | 0.93 | 0.03 (-0.66,0.73) | 0.92 |
|  |  | log additive: 0,1,2 | 1.22 (0.54,2.75) | 0.63 | 1.18 (0.62,2.25) | 0.61 | 0.00 (-0.39,0.39) | 1 |
| *ABCB1* | 2677G>T/A^##^  rs2032582 | codominant  TT vs GT vs GG | GT vs GG:  8.7 (1.21,62.3)  TT vs GG:  4.51 (0.47,43.51) | 0.038^+^ | GT vs GG:  1.37 (0.47,4.03)  TT vs GG:  0.84 (0.19,3.64) | 0.70 | GT vs GG:  -0.15 (-0.81,0.51)  TT vs GG:  -0.25 (-1.07,0.57) | 0.83 |
|  |  | dominant  GT + TT vs GG | 7.38 (1.07,51.05) | 0.016^+^ | 1.23 (0.43,3.47) | 0.70 | -0.18 (-0.80,0.44) | 0.57 |
|  |  | recessive:  TT vs GG + GT | 0.87 (0.2,3.83) | 0.85 | 0.68 (0.19,2.41) | 0.54 | -0.16 (-0.87,0.56) | 0.67 |
|  |  | log additive: 0,1,2 | 1.8 (0.75,4.29) | 0.18 | 0.97 (0.49,1.94) | 0.93 | -0.13 (-0.53,0.28) | 0.54 |
| *ABCB1* | 3435C>T  rs1045642 | codominant:  TT vs CT vs CC | CT vs CC:  4.63 (0.73,29.45)  TT vs CC:  6.11 (0.83,45.02) | 0.12 | CT vs CC:  2.78 (0.86,8.93)  TT vs CC:  0.74 (0.16,3.3) | 0.046^+^ | CT vs CC:  -0.45 (-1.13,0,22)  TT vs CC:  -0.24 (-1.01,0.54) | 0.42 |
|  |  | dominant:  CT + TT vs CC | 5.06 (0.84,30.52) | 0.043^+^ | 1.95 (0.63,6.02) | 0.24 | -0.38 (-1.01,0.25) | 0.24 |
|  |  | recessive:  TT vs CC + CT | 1.91 (0.55,6.67) | 0.31 | 0.36 (0.1,1.22) | 0.081 | 0.05 (-0.59,0.70) | 0.88 |
|  |  | log additive: 0,1,2 | 2.14 (0.91,5.04) | 0.071 | 0.9 (0.46,1.74) | 0.75 | -0.12 (-0.51,0.26) | 0.53 |
| *ABCB1* | TTT haplotype (1236,2677,3435) | codominant:  carriers vs non-carriers | 2.2 (0.6,8.06) | 0.22 | 1.62 (0.61,4.29) | 0.33 | -0.27 (-0.85,0.32) | 0.37 |
|  |  | dominant:  carriers vs non-carriers | 2.2 (0.6,8.06) | 0.22 | 1.62 (0.61,4.29) | 0.33 | -0.27 (-0.85,0.32) | 0.37 |
|  |  | log additive: 0,1,2 | 2.2 (0.6,8.06) | 0.22 | 1.62 (0.61,4.29) | 0.33 | -0.27 (-0.85,0.32) | 0.37 |

eGFR = estimated glomerular filtration rate at discharge, OR = Odds ratio, CI = Confidence Interval, Multivariate analysis includes HLA-mismatch <3 alleles vs. ≥3 alleles, % panel reactive antibodies: ≤ 10% (low risk) vs > 10% (high risk), AB0 compatibility: yes vs. no, previous transplantation: yes vs. no, living vs. deceased donor, basiliximab vs. thymoglobulin, valgancyclovir yes vs. no

^##^  the A allele was combined with T allele previous to statistical analysis, i.e. GA carriers were treated as GT carriers and TA carriers as TT carriers

§ not shown because only one homozygous carrier of minor allele was present in this cohort

+ not significant after correction for multiple testing

† according to Elens et al. 2013, Lloberas et al 2017

# square root transformed eGFR value

Supplementary Figure 1 A – D’ Plot for *ABCB1* variants


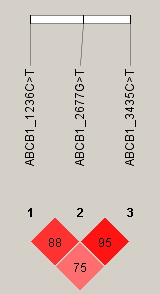


Supplementary Figure 1 B – r^2^ Plot for *ABCB1* variants


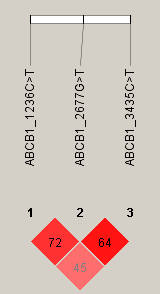

Supplement: Supplementary file 1 [file Table_1.docx]
